# Causal relationship between uterine fibroids and cardiovascular disease: A two-sample Mendelian randomization study

**DOI:** 10.1097/MD.0000000000041713

**Published:** 2025-02-28

**Authors:** Jie Cui, Yue-Chen Zhao, Li-Zhen She, Tie-Jun Wang

**Affiliations:** a Department of Radiation Oncology, The Second Hospital of Jilin University, Changchun, Jilin, China.

**Keywords:** cardiovascular disease, Mendelian randomization, single nucleotide polymorphism, uterine fibroids

## Abstract

Previous studies have indicated that patients with uterine fibroids (UF) may have an elevated risk of cardiovascular disease (CVD), although the causal relationship between UF and CVD remains unclear. In this Mendelian randomization (MR) study, we aimed to investigate the causal association between genetic susceptibility to UF and the risk of developing CVD. We extracted summary statistics for single nucleotide polymorphisms associated with UF and 5 CVDs from multiple databases for further analysis. First, we used linkage disequilibrium score regression to assess the genetic correlation across the genome. Next, we performed univariate MR (UVMR), and to ensure the robustness of our results, we conducted sensitivity analyses using several methods. Additionally, we applied multivariable MR (MVMR) to adjust for potential confounders. The linkage disequilibrium score regression results showed that there was no genetic correlation between UF and coronary heart disease, myocardial infarction (MI), atrial fibrillation, heart failure, cardioembolic stroke (CES). The UVMR revealed a significant association between UF and CES (OR = 1.113, 95% confidence interval [CI]: 1.018–1.218, *P* = .019, *P*_FDR_ = .047) and a suggestive causal relationship between UF and MI (OR = 0.943, 95% CI: 0.899–0.989, *P* = .015, *P*_FDR_ = .075). In the MVMR analysis, after adjusting for a range of potential confounders, the causal relationships between UF and both CES (OR = 1.104, 95% CI = 1.012–1.205, *P* = .027) and MI (OR = 0.935, 95% CI = 0.882–0.992, *P* = .025) remained significant. Our study found that UF increase the risk of CES but decrease the risk of MI, providing a theoretical basis for further research into the underlying mechanisms.

## 1. Introduction

Uterine fibroids (UF), also known as leiomyomas, are the most common benign tumors of the reproductive organs in women of reproductive age. They can affect the reproductive system in various ways, either individually or in combination. The most common initial symptom of fibroids is heavy menstrual bleeding, which can lead to anemia, fatigue, and dysmenorrhea. Other symptoms include noncyclical pain, abdominal distension, pain during intercourse, pelvic pressure, and bladder or bowel dysfunction, resulting in frequent urination, urinary retention, and constipation. UF may also be linked to reproductive issues, such as impaired fertility, pregnancy complications, and adverse obstetric outcomes, including an increased risk of preterm labor, cesarean section, antepartum hemorrhage, poor fetal development, and growth restriction.^[1,2]^ Statistically, between 5.4% and 77% of women are affected by leiomyomas, which are found in 70% of uteri after hysterectomy. In more than 80% of cases, multiple leiomyomas are present.^[3]^ Genetic factors, nulliparity, obesity, polycystic ovary syndrome, diabetes, and hypertension have all been shown to be associated with an increased risk of developing UF.^[4]^

Cardiovascular disease (CVD) is a group of conditions that affect the heart and blood vessels, including coronary artery disease, stroke, atrial fibrillation (AF), congestive heart failure (HF), and other heart-related disorders. As the leading cause of death worldwide, the prevalence of CVD nearly doubled between 1990 and 2019, rising from 271 million to 523 million cases. Similarly, the number of CVD-related deaths increased from 12.1 million to 18.6 million.^[5]^ Numerous studies have demonstrated a strong association between the presence of leiomyomas and the diagnosis of some form of CVD. An observational study found that women with a history of UF had a higher chance of developing CVD, including ischemic heart disease, HF, and stroke.^[6]^ Similarly, a cross-sectional study that included 104 patients with UF and 624 controls reported that women with UF had a significantly high risk of hypertension and more asymptomatic organ damage compared to the control group, especially in younger women.^[7]^ Yet another observational study found that the presence of UF was not associated with subclinical CVD, despite the fact that women with UF exhibited more CVD risk factors.^[8]^ Furthermore, traditional observational studies are subject to various confounding factors and reverse causality.^[9]^ Therefore, the potential causal relationship between UF and CVD remains unclear.

The Mendelian randomization (MR) approach uses genetic variation as an instrumental variable (IV) to provide evidence for a potential causal relationship between modifiable risk factors and disease.^[10]^ Genetic variants, typically single nucleotide polymorphisms (SNPs), are randomly distributed and independent of other traits, which helps minimize the impact of confounding factors and reverse causality.^[11]^ In the present study, we explored the causal relationship between UF and various CVDs through an MR study using summary statistics from genome-wide association studies (GWAS). Additionally, the linkage disequilibrium score regression (LDSC) method was used to quantitatively analyze the genetic correlations across the entire genome.

## 2. Materials and methods

### 2.1. Study design

**Figure 1.**
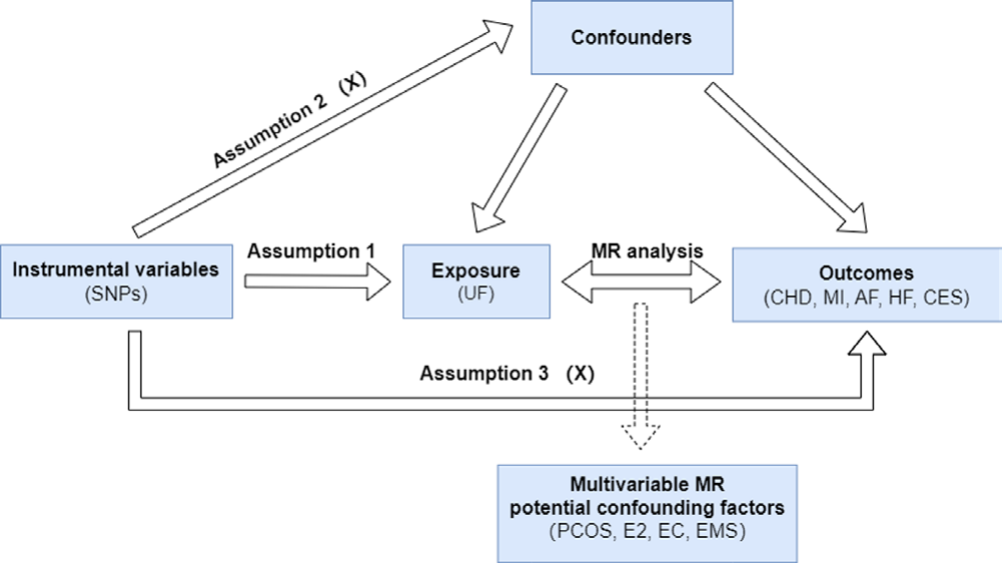
Study design flowchart of Mendelian randomization. AF = atrial fibrillation, CES = cardioembolic stroke, CHD = coronary heart disease, E2 = estradiol, EC = endometrial cancer, EMS = endometriosis, HF = heart failure, MI = myocardial infarction, MR = Mendelian randomization, PCOS = polycystic ovary syndrome, SNPs = single nucleotide polymorphisms, UF = uterine fibroids.

First, the genetic correlation between exposure and outcome was assessed using LDSC. This study then conducted univariable MR (UVMR) analysis assuming UF as the exposure variable and 5 CVDs including coronary heart disease (CHD), myocardial infarction (MI), AF, HF, and cardioembolic stroke (CES) as the outcome variables. The selection of IVs for UF was based on 3 key assumptions: (1) the genetic variation is strongly associated with exposure. (2) The genetic variant is not associated with any confounders of the exposure–outcome association. (3) The genetic variant affects the outcome only through its association with the exposure.^[12]^ To further explore the possibility of reverse causality, a reverse MR analysis was also performed. Since hypertension, diabetes, smoking, and sedentary behavior may act as confounders in the analysis of exposures versus outcomes, multivariable MR (MVMR) was applied to further explore the direct effect of exposures on outcomes (Fig. 1). It is important to note that all original studies included in this analysis received ethical approval, and participants provided informed consent. As a result, this study did not require additional ethical approval.

### 2.2. Genetic association datasets

#### 2.2.1. Genetic association dataset for UF

Genome-wide association studies (GWAS) associated with UF pooled data from the European Bioinformatics Institute database. To the best of our knowledge, this is the largest published GWAS meta-analysis of European pedigrees to date,^[13]^ which includes a total of 258,718 samples (21,024 cases and 237,694 controls) from the UK BioBank (UKB) and FinnGen. The UKB is a prospective, population-based study that recruited approximately 500,000 people in the United Kingdom with an average age between 40 and 69 years, and collected in-depth genetic and phenotypic data on them.^[14]^ As a public-private research project, FinnGen combines estimated genotype data generated from newly collected and legacy samples from the Finnish Biobank and digital health record data from the Finnish Health Registry, aiming to provide novel insights into medicine.^[15]^ The diagnostic criteria for UF were based on International Classification of Diseases (ICD)-10, with a specific code of D25.

#### 2.2.2. Genetic association dataset for 5 CVDs

We selected 5 CVDs as outcome variables for MR analysis, including CHD, MI, AF, HF, and CES. To increase the confidence of the analysis results, the GWAS summary data for the outcomes were obtained from databases other than UKB and FinnGenn. Additionally, we restricted the study population to Europeans to minimize possible pedigree mismatches. Pooled statistics for CHD were obtained from the Coronary ARtery DIsease Genome wide Replication and Meta-analysis (CARDIoGRAM) consortium, which conducted a meta-analysis of 14 GWAS for coronary artery disease, including 22,233 cases and 64,762 controls.^[16]^ Summary statistics for MI were from another GWAS analysis,^[17]^ including 14,825 cases and 2680 controls. Summary statistics for AF were from a GWAS meta-analysis of 6 studies that included 60,620 cases and 970,216 controls.^[18]^ For HF, summary statistics were derived from 47,309 cases and 930,014 controls from 26 studies.^[19]^ Finally, summary statistics for CES were obtained from the MEGASTROKE consortium, which includes 7193 cases and 406,111 controls.^[20]^ Detailed information is provided in Table 1. The diagnosis of CVD was based on ICD-10. CHD was defined as “ICD-10-I20, I21, I22”; MI as “ICD-10-I21, I22”; AF as “ICD-10-I48”; HF as “ICD-10-I50”; and CES as “ICD-10-I63.4.”

**Table 1:** Detailed information for the genome-wide association study data.

Traits	Data sources	GWAS ID	Sample size (cases/controls)	PMID	Population
Outcome					
CHD	Schunkert et al.	ieu-a-8	22,233/64,762	21378990	European
MI	Hartiala et al.	ebi-a-GCST011364	14,825/2680	33532862	European
AF	Nielsen et al.	ebi-a-GCST006414	60,620/970,216	30061737	European
HF	Shah et al.	ebi-a-GCST009541	47,308/930,014	31919418	European
CES	Malik et al.	ebi-a-GCST006910	7193/406,111	29531354	European
Exposure					
UF	Sakaue et al.	ebi-a-GCST90018934	21,024/237,694	34594039	European

AF = atrial fibrillation, CES = cardioembolic stroke, CHD = coronary heart disease, GWAS = genome-wide association studies, HF = heart failure, MI = myocardial infarction, PMID = PubMed identifier, UF = uterine fibroids.

### 2.3. Selection of IVs

We selected SNPs as IVs for the analysis. First, we extracted SNPs that were strongly correlated with exposure (*P* < 5.0 × 10^-8^). Second, to minimize bias caused by linkage disequilibrium (LD), the screened SNPs had to fulfill the following 2 conditions: *r*^2^ < 0.001, kb = 10,000. After going through these 2 steps, we recorded the resulting SNPs along with their variant identifier, effect size of allele, standard error of effect size, and *P*-value. To assess the strength of association between IVs and exposure factors, we used the F statistic. It was calculated as F = *R*^2^ (*N* - K - 1)/[K (1 - *R*^2^)]. Where *N* is the sample size of the exposed dataset, K is the number of SNPs, and *R*^2^ is the proportion of phenotypic variance explained by genetic variation. A value of F > 10 was considered sufficient to avoid bias from weak IVs.^[21]^

### 2.4. Statistical analysis

We harmonized SNP statistics for both the exposure and the outcomes to ensure that the allele assignments were consistent across all SNPs. Depending on the heterogeneity, an inverse variance weighted (IVW) approach with different models was used as the primary method of analysis. The IVW method relies on asymptotic estimates of the standard error of the causal (ratio) estimate from each variant, making it essential for each IV to be valid.^[22]^ At the same time, we used several other MR analysis methods, including weighted median, MR-Egger, simple mode, and weighted mode. The weighted median method provides consistent estimates of causal effects when up to half of the IVs are not valid.^[23]^ MR-Egger regression was used to detect and correct for the possible presence of pleiotropy.^[24]^ The simple mode and weighted mode methods were also applied as supplementary techniques to evaluate the causal effects of individual SNPs and create ensemble estimates.^[25]^ Furthermore, we applied the MR-PRESSO method to detect and remove potential outliers, ensuring more accurate MR analysis results.^[26]^ To correct for multiple comparisons in this study, we applied the Benjamini–Hochberg-based false discovery rate (FDR). The association between exposure and outcome was considered statistically significant if the IVW corrected *P*-value was <.05 and the results from the other methods were consistent in direction. An association was deemed suggestive if the initial IVW *P*-value was <.05, but the corrected *P*-value exceeded .05. Conversely, an association was considered unrelated if both the initial IVW *P*-value and the corrected *P*-value were >.05, with the corrected *P*-value being larger than the initial value.^[27]^ For causal associations with significant results in UVMR, we further performed MVMR to adjust for potential confounders, including hypertension, diabetes, smoking, and sedentary behavior. Sedentary behavior encompassed time spent using a computer and driving. The primary method of analysis was the IVW method.

Multiple sensitivity analyses were used in this study, with the *P*-values from all sensitivity tests compared against a significance threshold of .05. The intercept of MR-Egger regression was used to estimate the magnitude of horizontal pleiotropy.^[28]^ Cochran *Q*-value assessed the heterogeneity among individual SNPs. If the *P*-value was >.05, it indicated that there was no heterogeneity, and the IVW approach with a fixed-effects model was chosen; conversely, if the *P*-value was <.05, it indicated that there was heterogeneity, and the IVW approach with a random-effects model was chosen.^[29]^ Leave-one-out sensitivity analyses were performed to identify any individual SNPs that might have a disproportionate impact on the results. In addition, the results of the MR analyses were visualized using scatter plots, forest plots, and funnel plots. All statistical analyses were performed using the “TwoSampleMR” and “MR-PRESSO” packages in R software version 4.3.2, and all *P*-values were two-sided. MR estimates were represented by the odds ratio (OR) with 95% confidence intervals (CI).

### 2.5. Genetic correlation analysis

We employed the LDSC approach to assess genetic correlations between UF and CVD. Polygenicity as well as confounding bias may produce spurious distributions of test statistics in GWAS. LDSC allows us to detect the relationship between the test statistic and the LD to quantitatively analyze the effect of each component.^[30]^ In addition, LDSC can identify genetic correlations between complex traits and diseases to better explain etiology as well as prioritize possible causal relationships. This method is not biased by sample overlap.^[31]^ This method reflects whether the GWAS test statistic for a variant is associated with a nearby high chain imbalance variant by generating scores.^[32]^ The z-score for each variant in trait 1 was multiplied by the z-score for each variant in trait 2, and this product was regressed against the LD score to estimate genetic covariates. Genetic covariance normalized by SNP-heritability indicates genetic correlation.^[33]^

## 3. Results

### 3.1. Genetic correlation analysis

We applied the LDSC approach to assess the genetic correlation between UF and 5 CVDs. The results indicated that there was no genetic correlation between UF and CHD (*r*_g_ = 0.007, *P* = .933), MI (*r*_g_ = -0.068, *P* = .244), AF(*r*_g_ = -0.021, *P* = .665), HF (*r*_g_ = 0.233, *P* = .068), and CES (*r*_g_ = -0.011, *P* = .923).

### 3.2. Screening of IVs

We extracted SNPs strongly associated with UF from GWAS (*P* < 5.0 × 10^-8^). Afterward, we removed SNPs in LD (*r*^2^ < 0.001, kb = 10,000). Finally we excluded palindromic sequences with moderate allele frequencies. In total, 30 SNPs, 44 SNPs, 45 SNPs, 41 SNPs, and 45 SNPs were used to perform MR analyses of UF with CHD, MI, AF, HF, and CES, respectively. In addition, we did not observe any bias from weak IVs, as all F-statistics were >10 (Tables S1–S5, Supplemental Digital Content, http://links.lww.com/MD/O452).

### 3.3. Univariable MR analysis

**Figure 2.**
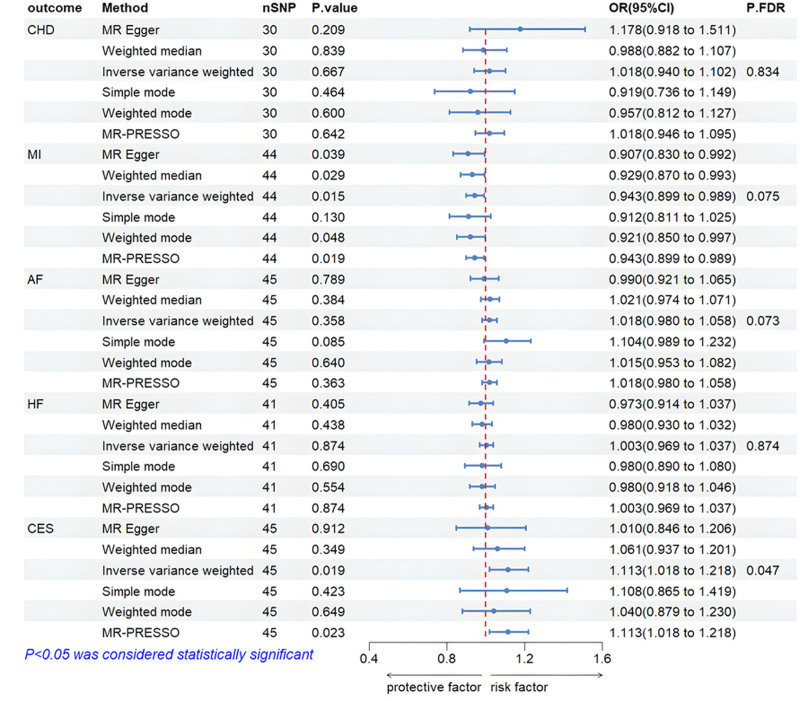
Mendelian randomization analysis of UF on the risk of CVD. AF = atrial fibrillation, CES = cardioembolic stroke, CHD = coronary heart disease, CI = confidence interval, FDR = false discovery rate, HF = heart failure, MI = myocardial infarction, OR = odds ratio, SNP = single nucleotide polymorphism.

The results, shown in Figure 2, indicate that the IVW approach in MR analysis revealed a significant positive correlation between genetically determined UF and CES (OR = 1.113, 95% CI: 1.018–1.218, *P* = .019, *P*_FDR_ = .047). The causal relationship between UF and MI was suggestive (OR = 0.943, 95% CI: 0.899–0.989, *P* = .015, *P*_FDR_ = .075). The causal relationship between UF and HF (OR = 1.003, 95% CI: 0.969–1.037, *P* = .874, *P*_FDR_ = .874), AF (OR = 1.018, 95% CI: 0.980–1.058, *P* = .358, *P*_FDR_ = .597), and CHD (OR = 1.018, 95% CI: 0.940–1.102, *P* = .667, *P*_FDR_ = .834) were not significantly associated. The scatterplot visualizing these results is shown in Figure S1, Supplemental Digital Content, http://links.lww.com/MD/O451. No outliers were identified using the MR-PRESSO method, suggesting that the analysis was reliable. In the test of heterogeneity, the Cochran Q statistic was >0.05 for all 4 CVDs except for UF and AF, where the Cochran Q statistic was <0.05. Therefore, except for AF, we used the IVW method with fixed-effects modeling as the main analysis method. Meanwhile, MR-Egger regression indicated that horizontal pleiotropy did not influence the causal relationship between UF and CVDs (Table 2). The leave-one-out method confirmed that no single SNP significantly impacted the causal relationship between UF and CVDs (Figure S2, Supplemental Digital Content, http://links.lww.com/MD/O451). Forest plots, shown in Figure S3, Supplemental Digital Content, http://links.lww.com/MD/O451 provided a visual representation of the effect size of individual SNPs and the overall analysis. Lastly, the funnel plots were roughly symmetrical, further supporting the absence of significant horizontal pleiotropy (Figure S4, Supplemental Digital Content, http://links.lww.com/MD/O451).

**Table 2:** Heterogeneity test and horizontal pleiotropy test.

Outcomes	Pleiotropy test			Heterogeneity test					
	MR-Egger			MR-Egger			Inverse variance weighted		
	Intercept	SE	*P*-val	Q	Q_df	Q_pval	Q	Q_df	Q_pval
Coronary heart disease	-0.012	0.010	.236	22.716	28	0.747	24.186	29	0.720
Myocardial infarction	0.004	0.004	.327	51.969	42	0.139	53.188	43	0.137
Atrial fibrillation	0.003	0.004	.376	87.392	43	7.38E-05	89.020	44	6.95E-05
Heart failure	0.003	0.003	.279	38.839	39	0.477	40.046	40	0.468
Cardioembolic stroke	0.010	0.008	.221	54.057	43	0.120	55.996	44	0.106

MR = Mendelian randomization, SE = standard error.

The results of reverse MR analysis indicated that CHD (OR = 0.997, 95% CI: 0.954–1.043, *P* = .905, *P*_FDR_ = .905), MI (OR = 1.018, 95% CI: 0.979–1.060, *P* = .371, *P*_FDR_ = .618), AF (OR = 1.014, 95% CI: 0.982–1.046, *P* = .399, *P*_FDR_ = .499), HF (OR = 0.883, 95% CI: 0.758–1.029, *P* = .111, *P*_FDR_ = .558), and CES (OR = 1.026, 95% CI: 0.976–1.079, *P* = .307, *P*_FDR_ = .769) were not significantly associated with the risk of UF (Table 3).

**Table 3:** Mendelian randomization analysis of cardiovascular diseases on the risk of uterine fibroids.

Exposure	Method	nSNP	OR	95% CI	*P*-value	*P*_FDR_
CHD	MR-Egger	15	0.981	0.856, 1.123	.783	
	Weighted median	15	1.018	0.963, 1.076	.538	
	Inverse variance weighted	15	0.997	0.954, 1.043	.905	.905
	Simple mode	15	1.006	0.900, 1.125	.916	
	Weighted mode	15	1.027	0.960, 1.098	.449	
	MR-PRESSO	15	0.997	0.954, 1.043	.907	
MI	MR-Egger	33	0.975	0.902, 1.055	.536	
	Weighted median	33	0.998	0.942, 1.058	.956	
	Inverse variance weighted	33	1.018	0.979, 1.060	.371	.618
	Simple mode	33	1.017	0.907, 1.139	.780	
	Weighted mode	33	1.008	0.936, 1.085	.838	
	MR-PRESSO	33	1.018	0.979, 1.060	.377	
AF	MR Egger	111	1.048	0.988, 1.112	.120	
	Weighted median	111	1.025	0.977, 1.075	.312	
	Inverse variance weighted	111	1.014	0.982, 1.046	.399	.499
	Simple mode	111	1.062	0.965, 1.169	.223	
	Weighted mode	111	1.036	0.995, 1.080	.092	
	MR-PRESSO	111	1.016	0.986, 1.048	.291	
HF	MR-Egger	9	0.870	0.577, 1.312	.529	
	Weighted median	9	0.897	0.774, 1.040	.149	
	Inverse variance weighted	9	0.883	0.758, 1.029	.112	.558
	Simple mode	9	0.861	0.673, 1.100	.266	
	Weighted mode	9	0.900	0.761, 1.065	.256	
	MR-PRESSO	9	0.883	0.758, 1.029	.150	
CES	MR Egger	9	0.976	0.870, 1.094	.714	
	Weighted median	9	1.022	0.968, 1.078	.438	
	Inverse variance weighted	9	1.026	0.976, 1.079	.307	.769
	Simple mode	9	1.008	0.937, 1.086	.840	
	Weighted mode	9	1.021	0.959, 1.086	.564	
	MR-PRESSO	9	1.026	0.991, 1.063	.245	

AF = atrial fibrillation, CES = cardioembolic stroke, CHD = coronary heart disease, CI = confidence interval, FDR = false discovery rate, HF = heart failure, MI = myocardial infarction, OR = odds ratio, SNP = single nucleotide polymorphism.

### 3.4. Multivariable MR analysis

Considering that hypertension, diabetes, smoking, and sedentary behavior may act as confounders affecting the causal analysis between UF and CVD, we applied the MVMR method to explore the direct effect of UF on the relationship with various CVD. The IVW analysis results (Table 4) indicated a significant causal association between UF and both CES (OR = 1.104, 95% CI = 1.012–1.205, *P* = .027) and MI (OR = 0.935, 95% CI = 0.882–0.992, *P* = .025), consistent with the findings from the UVMR analysis. Furthermore, after adjusting for confounders, no significant causal associations were found between UF and CHD, AF, or HF. Meanwhile, we performed heterogeneity tests, and the results indicated that there was no heterogeneity for CHD (Q = 135, *P* = .051), HF (Q = 165, *P* = .060), and CES (Q = 181, *P* = .080), whereas heterogeneity was present for MI (Q = 283, *P* < .05) and AF (Q = 135, *P* < .05).

**Table 4:** Results of multivariate Mendelian randomization analysis.

Outcome	Exposure	OR	95% CI	*P*-value
CHD	Diabetes	3.603	1.087, 11.945	.036
	Time spent using computer	0.583	0.386, 0.880	.010
	Time spent driving	2.504	0.952, 6.582	.063
	Smoking/smokers in household	0.238	0.007, 7.744	.419
	Hypertension	5.676	0, 2.456E + 09	.864
	Uterine fibroids	1.016	0.928, 1.111	.737
MI	Diabetes	4.064	1.549, 10.667	.004
	Time spent using computer	0.856	0.617, 1.189	.355
	Time spent driving	1.019	0.454, 2.291	.963
	Smoking/smokers in household	8.047	0.546, 118.610	.129
	Hypertension	4.333E + 06	2.177, 8.624E + 12	.039
	Uterine fibroids	0.935	0.882, 0.992	.025
AF	Diabetes	0.428	0.218, 0.839	.013
	Time spent using computer	0.922	0.737, 1.154	.479
	Time spent driving	1.969	1.140, 3.402	.015
	Smoking/smokers in household	3.335	0.516, 21.545	.206
	Hypertension	4.156E + 07	1.553E + 03, 1.112E + 12	.001
	Uterine fibroids	0.998	0.958, 1.039	.923
HF	Diabetes	0.721	0.397, 1.312	.285
	Time spent using computer	0.906	0.740, 1.108	.336
	Time spent driving	1.481	0.907, 2.421	.117
	Smoking/smokers in household	1.209	0.234, 6.239	.821
	Hypertension	4.044	0, 34857.379	.762
	Uterine fibroids	0.994	0.958, 1.032	.758
CES	Diabetes	0.544	0.128, 2.322	.411
	Time spent using computer	0.988	0.615, 1.587	.961
	Time spent driving	1.907	0.600, 6.057	.274
	Smoking/smokers in household	0.144	0.003, 7.062	.339
	Hypertension	7.644E + 08	0.2025, 2.856E + 18	.069
	Uterine fibroids	1.104	1.012, 1.205	.027

AF = atrial fibrillation, CES = cardioembolic stroke, CHD = coronary heart disease, CI = confidence interval, HF = heart failure, MI = myocardial infarction, OR = odds ratio.

## 4. Discussion

For the first time, we systematically investigated the potential causal relationship between genetic susceptibility to UF and the risk of CVDs at the genetic level using MR methods. Our findings revealed a significant positive association between genetic susceptibility to UF and the risk of CES. The causal relationship between UF and MI was suggestive, but not definitive. There was insufficient evidence to support a causal association between UF and CHD, AF, and HF. After adjusting for potential confounders, associations between UF and both MI and CES were still observed. Specifically, UF was positively correlated with CES and negatively correlated with MI. Additionally, reverse MR analysis demonstrated no causal relationships between CHD, MI, AF, HF, CES, and UF.

Although both men and women share several common risk factors for CVD, women also have unique risk factors, such as the use of oral contraceptives.^[34]^ Various gestational conditions, including gestational hypertension, diabetes, and preeclampsia, may also contribute to the development of CVD in women. In addition, polycystic ovary syndrome as well as early menopause have been associated with an increased risk of CVD.^[35,36]^ In recent years, there has been growing interest in the potential role of UF as a new risk factor for CVD in women. One hypothesis suggests that UF and CVD share similarities in their processes of wound healing or arterial plaque formation. Through these mechanisms, the synthesis of extracellular matrix in UF or the formation of arterial plaques may be accelerated, contributing to both UF and the hypertrophy of ventricular and vascular walls.^[8]^ Moreover, an association has been observed between UF and several known CVD risk factors, including hypertension, obesity, serum lipid abnormalities, and carotid intima-media thickness, which may further increase the risk of CVD.^[37]^ However, in our study, we did not find significant associations between UF and CHD, AF, or HF. This lack of association may be attributed to confounding factors inherent in observational studies. Furthermore, CVD endpoints in women with UF remain understudied. For example, hysterectomy is the mainstay of treatment for UF, and studies have reported an increased risk of CVD after hysterectomy.^[38]^ This could have influenced the study results. Additionally, given that UF primarily affects women of reproductive age, and the risk of CVD in postmenopausal women increases over time, it is important to follow women with UF over extended periods, taking into account the normal progression of CVD.^[8]^

UF are common in women, but ischemic stroke, particularly CES, is not a frequent complication associated with UF. Several case reports have described instances of ischemic stroke in patients with UF. For example, Higuchi et al reported a case of recurrent ischemic stroke in a middle-aged woman with UF, and suggested that UF may cause hypercoagulability in this patient, as evidenced by elevated plasma D-dimer levels and ultrasound Doppler findings of microembolic signals in the right middle cerebral artery.^[39]^ A multicenter retrospective study from Japan found that 39 of 470 female patients with ischemic stroke or transient ischemic attack had common noncancerous gynecologic diseases, including 24 patients (62%) with UF. They were further found to have cryptogenic strokes (predominantly due to CES), nonbacterial thrombotic endocarditis, and paradoxical embolism. These conditions were largely attributed to the development of hypercoagulable states, with significantly elevated levels of CA125 and D-dimer observed in these patients.^[40]^ CA125 is a mucin molecule and serves as a serum marker.^[41]^ Mucins can promote thrombosis through adhesion-dependent interactions between neutrophils and platelets, as well as the bidirectional signaling pathway between them.^[42]^ A retrospective study by Buamah, conducted over a period of 42 months, revealed elevated serum levels of CA125 in 6 patients with nonmalignant gynecological diseases, 2 of whom had UF.^[43]^ However, some studies have indicated that CA125 is not associated with UF. For instance, Dawood et al conducted a prospective study comparing the serologic indices of 51 women with UF at more than 14 weeks of gestation with those of 30 normal childbearing women, and the results showed no difference in CA125 levels during the follicular and luteal phases between the 2 groups.^[44]^ D-dimer, a marker of thrombin formation and fibrinolysis, can indicate thrombus formation.^[45]^ Recent studies have found that high high D-dimer levels may point to an undetected source of cardiac embolism, such as silent AF, as the etiology of stroke in some patients.^[46]^ In conclusion, we found a causal relationship between UF and CES, providing a theoretical basis for the early diagnosis and treatment of such patients. However, the underlying mechanisms linking UF and CES remain poorly understood. Future research should focus on exploring the potential pathogenesis of CES in patients with UF, which may facilitate timely clinical intervention.

Interestingly, our study found a potential association between UF and MI, with UF appearing to reduce the risk of MI. This phenomenon may be closely related to the hormone levels in women. Estrogen and progesterone play significant roles in the development and progression of UF. They can stimulate mature leiomyoma cells to release proliferative signals, thereby promoting the transformation of surrounding undifferentiated cells into those that may support tumor growth. Progesterone is essential for the proliferation of uterine fibroid cells, while estrogen enhances cell sensitivity to progesterone by increasing the availability of progesterone receptors.^[47]^ Studies have shown that estrogen induces rapid dilation of coronary arteries, offering vascular protective effects. Additionally, estrogen can exert anti-inflammatory effects by reducing the expression of neutrophil chemoattractant factor (CINC-2β) and monocyte chemoattractant protein (MCP-1) and inhibiting the expression of C-reactive protein.^[48]^ Korkmaz et al, in their prospective studies, explored the risk of CVD in UF patients and found that although the number of UF correlates positively with triglycerides and LDL levels, the intima-media thickness of the carotid artery in UF patients was significantly lower, which may be related to the vascular protective effects of estrogen.^[49]^ Although existing studies suggest a potential role of hormones in the relationship between UF and MI, further exploration of other potential mechanisms is needed to better understand the impact of UF on MI.

To the best of our knowledge, this study is the first MR analysis to explore the causal association between UF and CVD. By utilizing data from a large-scale GWAS, we were able to expand the association analysis between UF and CVD at the genetic level, while avoiding the limitations inherent in traditional observational studies. The application of multiple sensitivity analysis methods further strengthened the robustness and reliability of our findings. Additionally, the MR approach benefits from the process of randomization, which enhances its methodological rigor while also being time- and cost-efficient.

Our study has several limitations. First, the MR approach did not allow for the assessment of a potential nonlinear relationship between UF and CVD, and the absence of clinical data prevented us from conducting further subgroup analyses. Second, our study was limited to populations of European ancestry, which may introduce bias due to the homogeneous nature of the sample, thereby limiting the generalizability of the findings to other populations. Third, despite efforts to mitigate pleiotropic effects, some potential pleiotropy could not be excluded, as the biological functions of many genetic variants remain unclear. Finally, while we have made a preliminary assessment of the causal relationship between UF and CVD, the exact mechanisms underlying their interaction remain unclear and warrant further investigation.

## 5. Conclusion

Our study provides evidence for a potential causal effect between UF and CES as well as MI, laying the foundation for further mechanistic research. However, we did not find a causal relationship between UF and CHD, AF, or HF. Further research is needed to validate our findings and ultimately draw more conclusive results.

## Author contributions

**Conceptualization:** Jie Cui, Yue-Chen Zhao, Tie-Jun Wang.

**Formal analysis:** Li-Zhen She.

**Methodology:** Jie Cui.

**Writing – original draft:** Jie Cui.

**Writing – review & editing:** Yue-Chen Zhao, Li-Zhen She, Tie-Jun Wang.

## Supplementary Material



## References

[1] Vercellini P Frattaruolo MP. Uterine fibroids: from observational epidemiology to clinical management. BJOG. 2017;124:1513. 28498567 10.1111/1471-0528.14730

[2] Giuliani E As-Sanie S Marsh EE. Epidemiology and management of uterine fibroids. Int J Gynaecol Obstet. 2020;149:3–9. 31960950 10.1002/ijgo.13102

[3] Sparic R Mirkovic L Malvasi A Tinelli A. Epidemiology of uterine myomas: a review. Int J Fertil Steril. 2016;9:424–35. 26985330 10.22074/ijfs.2015.4599 PMC4793163

[4] Okolo S. Incidence, aetiology and epidemiology of uterine fibroids. Best Pract Res Clin Obstet Gynaecol. 2008;22:571–88. 18534913 10.1016/j.bpobgyn.2008.04.002

[5] Li Y Cao GY Jing WZ Liu J Liu M. Global trends and regional differences in incidence and mortality of cardiovascular disease, 1990-2019: findings from 2019 global burden of disease study. Eur J Prev Cardiol. 2023;30:276–86. 36458973 10.1093/eurjpc/zwac285

[6] Brewster LM Haan Y van Montfrans GA. Cardiometabolic risk and cardiovascular disease in young women with uterine fibroids. Cureus. 2022;14:e30740. 36447683 10.7759/cureus.30740 PMC9699995

[7] Haan YC Diemer FS Van Der Woude L Van Montfrans GA Oehlers GP Brewster LM. The risk of hypertension and cardiovascular disease in women with uterine fibroids. J Clin Hypertens (Greenwich). 2018;20:718–26. 29569360 10.1111/jch.13253 PMC8030762

[8] Laughlin-Tommaso SK Fuchs EL Wellons MF. Uterine fibroids and the risk of cardiovascular disease in the coronary artery risk development in young adult women’s study. J Womens Health (Larchmt). 2019;28:46–52. 30412447 10.1089/jwh.2018.7122 PMC6343187

[9] Hammerton G Munafò MR. Causal inference with observational data: the need for triangulation of evidence. Psychol Med. 2021;51:563–78. 33682654 10.1017/S0033291720005127 PMC8020490

[10] Davies NM Holmes MV Davey Smith G. Reading Mendelian randomisation studies: a guide, glossary, and checklist for clinicians. BMJ. 2018;362:k601. 30002074 10.1136/bmj.k601 PMC6041728

[11] Power GM Sanderson E Pagoni P. Methodological approaches, challenges, and opportunities in the application of Mendelian randomisation to lifecourse epidemiology: a systematic literature review. Eur J Epidemiol. 2024;39:501–20. 37938447 10.1007/s10654-023-01032-1 PMC7616129

[12] Davey Smith G Hemani G. Mendelian randomization: genetic anchors for causal inference in epidemiological studies. Hum Mol Genet. 2014;23:R89–98. 25064373 10.1093/hmg/ddu328 PMC4170722

[13] Sakaue S Kanai M Tanigawa Y.; FinnGen. A cross-population atlas of genetic associations for 220 human phenotypes. Nat Genet. 2021;53:1415–24. 34594039 10.1038/s41588-021-00931-x PMC12208603

[14] Bycroft C Freeman C Petkova D. The UK Biobank resource with deep phenotyping and genomic data. Nature. 2018;562:203–9. 30305743 10.1038/s41586-018-0579-z PMC6786975

[15] Kurki MI Karjalainen J Palta P. FinnGen provides genetic insights from a well-phenotyped isolated population. Nature. 2023;613:508–18. 36653562 10.1038/s41586-022-05473-8 PMC9849126

[16] Schunkert H König IR Kathiresan S. Large-scale association analysis identifies 13 new susceptibility loci for coronary artery disease. Nat Genet. 2011;43:333–8. 21378990 10.1038/ng.784 PMC3119261

[17] Hartiala JA Han Y Jia Q. Genome-wide analysis identifies novel susceptibility loci for myocardial infarction. Eur Heart J. 2021;42:919–33. 33532862 10.1093/eurheartj/ehaa1040 PMC7936531

[18] Nielsen JB Thorolfsdottir RB Fritsche LG. Biobank-driven genomic discovery yields new insight into atrial fibrillation biology. Nat Genet. 2018;50:1234–9. 30061737 10.1038/s41588-018-0171-3 PMC6530775

[19] Shah S Henry A Roselli C. Genome-wide association and Mendelian randomisation analysis provide insights into the pathogenesis of heart failure. Nat Commun. 2020;11:163. 31919418 10.1038/s41467-019-13690-5 PMC6952380

[20] Malik R Chauhan G Traylor M. Multiancestry genome-wide association study of 520,000 subjects identifies 32 loci associated with stroke and stroke subtypes. Nat Genet. 2018;50:524–37. 29531354 10.1038/s41588-018-0058-3 PMC5968830

[21] Burgess S Thompson SG. Avoiding bias from weak instruments in Mendelian randomization studies. Int J Epidemiol. 2011;40:755–64. 21414999 10.1093/ije/dyr036

[22] Burgess S Butterworth A Thompson SG. Mendelian randomization analysis with multiple genetic variants using summarized data. Genet Epidemiol. 2013;37:658–65. 24114802 10.1002/gepi.21758 PMC4377079

[23] Bowden J Davey Smith G Haycock PC Burgess S. Consistent estimation in Mendelian randomization with some invalid instruments using a weighted median estimator. Genet Epidemiol. 2016;40:304–14. 27061298 10.1002/gepi.21965 PMC4849733

[24] Burgess S Thompson SG. Interpreting findings from Mendelian randomization using the MR-Egger method. Eur J Epidemiol. 2017;32:377–89. 28527048 10.1007/s10654-017-0255-x PMC5506233

[25] Gao Y Fan ZR Shi FY. Hypothyroidism and rheumatoid arthritis: a two-sample Mendelian randomization study. Front Endocrinol (Lausanne). 2023;14:1179656. 37324262 10.3389/fendo.2023.1179656 PMC10262846

[26] Verbanck M Chen CY Neale B Do R. Detection of widespread horizontal pleiotropy in causal relationships inferred from Mendelian randomization between complex traits and diseases. Nat Genet. 2018;50:693–8. 29686387 10.1038/s41588-018-0099-7 PMC6083837

[27] Chen J Yuan S Fu T. Gastrointestinal consequences of type 2 diabetes mellitus and impaired glycemic homeostasis: a Mendelian randomization study. Diabetes Care. 2023;46:828–35. 36800530 10.2337/dc22-1385 PMC10091506

[28] Bowden J Davey Smith G Burgess S. Mendelian randomization with invalid instruments: effect estimation and bias detection through Egger regression. Int J Epidemiol. 2015;44:512–25. 26050253 10.1093/ije/dyv080 PMC4469799

[29] Gao N Kong M Li X. Systemic lupus erythematosus and cardiovascular disease: a Mendelian randomization study. Front Immunol. 2022;13:908831. 35734181 10.3389/fimmu.2022.908831 PMC9207262

[30] Bulik-Sullivan BK Loh PR Finucane HK. LD Score regression distinguishes confounding from polygenicity in genome-wide association studies. Nat Genet. 2015;47:291–5. 25642630 10.1038/ng.3211 PMC4495769

[31] Bulik-Sullivan B Finucane HK Anttila V. An atlas of genetic correlations across human diseases and traits. Nat Genet. 2015;47:1236–41. 26414676 10.1038/ng.3406 PMC4797329

[32] Wielscher M Amaral AFS van der Plaat D. Genetic correlation and causal relationships between cardio-metabolic traits and lung function impairment. Genome Med. 2021;13:104. 34154662 10.1186/s13073-021-00914-x PMC8215837

[33] Cui G Li S Ye H. Gut microbiome and frailty: insight from genetic correlation and mendelian randomization. Gut Microbes. 2023;15:2282795. 37990415 10.1080/19490976.2023.2282795 PMC10730212

[34] Baillargeon JP McClish DK Essah PA Nestler JE. Association between the current use of low-dose oral contraceptives and cardiovascular arterial disease: a meta-analysis. J Clin Endocrinol Metab. 2005;90:3863–70. 15814774 10.1210/jc.2004-1958

[35] Appelman Y van Rijn BB Ten Haaf ME Boersma E Peters SA. Sex differences in cardiovascular risk factors and disease prevention. Atherosclerosis. 2015;241:211–8. 25670232 10.1016/j.atherosclerosis.2015.01.027

[36] Haan YCL. Cardiovascular risk in women with uterine fibroids of different ethnic groups. Universiteit van Amsterdam Press; 2020:12.

[37] Uimari O Auvinen J Jokelainen J. Uterine fibroids and cardiovascular risk. Hum Reprod. 2016;31:2689–703. 27733532 10.1093/humrep/dew249

[38] Wang Z Wu J Zhang D. Hysterectomy and ischemic heart disease: An observational study using propensity score methods in NHANES 2007-2018. Atherosclerosis. 2021;327:5–12. 34004485 10.1016/j.atherosclerosis.2021.04.009

[39] Higuchi E Toi S Shirai Y. Recurrent cerebral infarction due to benign uterine myoma. J Stroke Cerebrovasc Dis. 2019;28:e1–2. 30366865 10.1016/j.jstrokecerebrovasdis.2018.09.034

[40] Yamashiro K Sato T Nito C. Stroke in patients with common noncancerous gynecologic diseases: a multicenter study in Japan. Neurol Clin Pract. 2023;13:e200165. 37124460 10.1212/CPJ.0000000000200165 PMC10140918

[41] Yin BW Lloyd KO. Molecular cloning of the CA125 ovarian cancer antigen: identification as a new mucin, MUC16. J Biol Chem. 2001;276:27371–5. 11369781 10.1074/jbc.M103554200

[42] Shao B Wahrenbrock MG Yao L. Carcinoma mucins trigger reciprocal activation of platelets and neutrophils in a murine model of Trousseau syndrome. Blood. 2011;118:4015–23. 21860019 10.1182/blood-2011-07-368514 PMC3204725

[43] Buamah P. Benign conditions associated with raised serum CA-125 concentration. J Surg Oncol. 2000;75:264–5. 11135268 10.1002/1096-9098(200012)75:4<264::aid-jso7>3.0.co;2-q

[44] Dawood MY Khan-Dawood FS. Plasma insulin-like growth factor-I, CA-125, estrogen, and progesterone in women with leiomyomas. Fertil Steril. 1994;61:617–21. 8150101 10.1016/s0015-0282(16)56635-7

[45] Bates SM. D-dimer assays in diagnosis and management of thrombotic and bleeding disorders. Semin Thromb Hemost. 2012;38:673–82. 23041982 10.1055/s-0032-1326782

[46] Ohara T Farhoudi M Bang OY Koga M Demchuk AM. The emerging value of serum D-dimer measurement in the work-up and management of ischemic stroke. Int J Stroke. 2020;15:122–31. 31537182 10.1177/1747493019876538

[47] Reis FM Bloise E Ortiga-Carvalho TM. Hormones and pathogenesis of uterine fibroids. Best Pract Res Clin Obstet Gynaecol. 2016;34:13–24. 26725037 10.1016/j.bpobgyn.2015.11.015

[48] Xing D Nozell S Chen YF Hage F Oparil S. Estrogen and mechanisms of vascular protection. Arterioscler Thromb Vasc Biol. 2009;29:289–95. 19221203 10.1161/ATVBAHA.108.182279 PMC2700771

[49] Korkmaz V Ozkaya E Özer Kadife S Kara F Kucukozkan T. Investigation of cardiovascular disease risk in women with uterine leiomyomas. Ir J Med Sci. 2016;185:689–93. 26208583 10.1007/s11845-015-1343-0

