# Supplementary material for: Causal relationship between uterine fibroids and cardiovascular disease: A two-sample Mendelian randomization study
Source: Medicine (Baltimore). 2025 Feb 28;104(9):e41713. doi: 10.1097/MD.0000000000041713 (PMC11875593; doi:10.1097/MD.0000000000041713)
Supplement: Supplementary file 1 [file medi-104-e41713-s001.pdf]

**Supplementary Table 1.** Detailed information of SNPs used in the MR analysis of UF on CHD.

| SNP | Chr | Position | A1 | A2 | EAF | Uterine Fibroids | | | Coronary Heart Disease | | | F-val |
|---|---|---|---|---|---|---|---|---|---|---|---|---|
| | | | | | | Beta | SE | P-val | Beta | SE | P-val | |
| rs10069690 | 5 | 1279790 | T | C | 0.262543 | 0.1226 | 0.01 | 1.79E-34 | -0.02 | 0.033 | 0.544473 | 150.3064 |
| rs1045405 | 11 | 252733 | C | T | 0.152915 | 0.0759 | 0.0127 | 2.07E-09 | -0.06114 | 0.047371 | 0.196816 | 35.71682 |
| rs10815717 | 9 | 804924 | A | G | 0.495339 | 0.0776 | 0.009 | 6.76E-18 | -0.00291 | 0.014004 | 0.835621 | 74.34214 |
| rs10947616 | 6 | 36623083 | G | C | 0.273529 | -0.0695 | 0.0096 | 5.86E-13 | -0.00104 | 0.017339 | 0.952033 | 52.41116 |
| rs11008551 | 10 | 31913890 | T | G | 0.197621 | -0.0609 | 0.011 | 3.03E-08 | 0.008848 | 0.021384 | 0.679036 | 30.65109 |
| rs11786929 | 8 | 1.3E+08 | C | T | 0.365194 | 0.0544 | 0.009 | 1.53E-09 | 0.002889 | 0.016879 | 0.864095 | 36.53503 |
| rs11887109 | 2 | 1E+08 | G | A | 0.525423 | 0.0593 | 0.0088 | 1.56E-11 | 0.023403 | 0.015312 | 0.126412 | 45.40887 |
| rs12479436 | 2 | 11666339 | G | T | 0.585117 | 0.0839 | 0.0087 | 8.06E-22 | -0.00738 | 0.014507 | 0.610793 | 92.99981 |
| rs12674731 | 8 | 30175381 | C | A | 0.27411 | 0.0574 | 0.0095 | 1.49E-09 | -0.00954 | 0.018301 | 0.602304 | 36.50675 |
| rs12902948 | 15 | 77850146 | C | T | 0.279137 | -0.0629 | 0.01 | 3.50E-10 | -0.01716 | 0.015084 | 0.255345 | 39.56379 |
| rs1506869 | 8 | 25269102 | A | C | 0.328855 | -0.052 | 0.0091 | 1.02E-08 | -0.02116 | 0.015871 | 0.182451 | 32.65281 |
| rs16991615 | 20 | 5948227 | A | G | 0.05205 | 0.1451 | 0.0265 | 4.30E-08 | 0.021693 | 0.030012 | 0.469793 | 29.98056 |
| rs17033114 | 12 | 1.03E+08 | C | T | 0.125296 | -0.1322 | 0.0145 | 7.35E-20 | -0.03942 | 0.03851 | 0.306033 | 83.12345 |
| rs17116149 | 10 | 1.06E+08 | A | G | 0.038813 | 0.2688 | 0.0197 | 1.83E-42 | 0.113656 | 0.079994 | 0.155372 | 186.1756 |
| rs17631680 | 2 | 67090367 | C | T | 0.101614 | -0.1045 | 0.0179 | 5.14E-09 | -0.03288 | 0.023981 | 0.170395 | 34.08185 |
| rs2131371 | 12 | 46796522 | C | A | 0.731473 | 0.075 | 0.0101 | 1.20E-13 | 0.010181 | 0.014534 | 0.483617 | 55.14123 |
| rs2207548 | 11 | 32368744 | A | C | 0.323407 | 0.1026 | 0.0103 | 1.38E-23 | 0.023336 | 0.014362 | 0.104192 | 99.22404 |
| rs2277339 | 12 | 57146069 | G | T | 0.132493 | -0.0801 | 0.0121 | 3.12E-11 | 0.008797 | 0.028318 | 0.756082 | 43.82187 |
| rs2293607 | 3 | 1.69E+08 | C | T | 0.343688 | -0.0845 | 0.0093 | 1.61E-19 | 0.026156 | 0.016459 | 0.112033 | 82.55515 |
| rs2306022 | 15 | 68628163 | T | C | 0.08676 | 0.1104 | 0.0152 | 3.89E-13 | 0.0176 | 0.026026 | 0.498894 | 52.75305 |
| rs2413620 | 22 | 40668988 | G | C | 0.256586 | 0.1028 | 0.0095 | 4.35E-27 | -0.00158 | 0.016721 | 0.924516 | 117.0943 |
| rs34933909 | 5 | 1.76E+08 | T | G | 0.459616 | 0.0916 | 0.0151 | 1.44E-09 | -0.01235 | 0.014091 | 0.38069 | 36.7988 |
| rs3804984 | 3 | 4716214 | C | T | 0.470262 | -0.0618 | 0.0092 | 1.89E-11 | -0.0016 | 0.014429 | 0.911681 | 45.123 |
| rs3820282 | 1 | 22468215 | T | C | 0.250518 | 0.1542 | 0.0102 | 4.06E-52 | -0.0014 | 0.019267 | 0.942235 | 228.5415 |
| rs547904 | 3 | 8947601 | G | A | 0.657141 | 0.0505 | 0.0092 | 4.86E-08 | -0.01311 | 0.015468 | 0.396592 | 30.13032 |
| rs6903575 | 6 | 74508038 | A | G | 0.495939 | -0.0618 | 0.0085 | 4.50E-13 | 0.003538 | 0.013924 | 0.799439 | 52.86104 |
| rs7043754 | 9 | 652079 | A | G | 0.443614 | -0.0542 | 0.0087 | 4.75E-10 | 0.009405 | 0.014135 | 0.505799 | 38.81117 |

| | | | | | | | | | | | | |
|---|---|---|---|---|---|---|---|---|---|---|---|---|
| rs7841047 | 8 | 71265978 | A | G | 0.247413 | 0.0651 | 0.0102 | 1.96E-10 | -0.01723 | 0.019296 | 0.371894 | 40.73411 |
| rs8105767 | 19 | 22215441 | G | A | 0.311329 | 0.0496 | 0.0091 | 4.97E-08 | -0.01939 | 0.015522 | 0.211611 | 29.70826 |
| rs9549260 | 13 | 41254104 | A | C | 0.284865 | 0.0559 | 0.0092 | 1.53E-09 | -0.00521 | 0.016914 | 0.757923 | 36.91855 |

SNP, single nucleotide polymorphism; Chr, chromosome; EAF, effect allele frequency.

**Supplementary Table 2.** Detailed information of SNPs used in the MR analysis of UF on MI.

| SNP | Chr | Position | A1 | A2 | EAF | Uterine Fibroids | | | Myocardial Infarction | | | *F*-val |
|---|---|---|---|---|---|---|---|---|---|---|---|---|
| | | | | | | Beta | SE | *P*-val | Beta | SE | *P*-val | |
| rs10069690 | 5 | 1279790 | T | C | 0.262543 | 0.1226 | 0.01 | 1.79E-34 | -0.01215 | 0.012212 | 0.32 | 150.3064 |
| rs1045405 | 11 | 252733 | C | T | 0.152915 | 0.0759 | 0.0127 | 2.07E-09 | 0.001646 | 0.027733 | 0.95 | 35.71682 |
| rs10815717 | 9 | 801571 | A | G | 0.495339 | 0.0776 | 0.009 | 6.76E-18 | -0.01731 | 0.01112 | 0.12 | 74.34214 |
| rs10947616 | 6 | 36620499 | G | C | 0.273529 | -0.0695 | 0.0096 | 5.86E-13 | 0.014453 | 0.012944 | 0.26 | 52.41116 |
| rs11008551 | 10 | 31913890 | T | G | 0.197621 | -0.0609 | 0.011 | 3.03E-08 | 0.02286 | 0.013559 | 0.092001 | 30.65109 |
| rs117245733 | 13 | 40723944 | A | G | 0.020552 | 0.3827 | 0.0379 | 6.12E-24 | -0.02927 | 0.044959 | 0.52 | 101.9612 |
| rs11786929 | 8 | 1.3E+08 | C | T | 0.365194 | 0.0544 | 0.009 | 1.53E-09 | 0.01131 | 0.011456 | 0.32 | 36.53503 |
| rs11887109 | 2 | 1E+08 | G | A | 0.525423 | 0.0593 | 0.0088 | 1.56E-11 | 0.006626 | 0.010835 | 0.54 | 45.40887 |
| rs12479436 | 2 | 11666339 | G | T | 0.585117 | 0.0839 | 0.0087 | 8.06E-22 | -0.01066 | 0.010983 | 0.33 | 92.99981 |
| rs12674731 | 8 | 30175381 | C | A | 0.27411 | 0.0574 | 0.0095 | 1.49E-09 | 0.003399 | 0.012758 | 0.79 | 36.50675 |
| rs12902948 | 15 | 77849254 | C | T | 0.279137 | -0.0629 | 0.01 | 3.50E-10 | -0.00293 | 0.011616 | 0.8 | 39.56379 |
| rs149934734 | 11 | 1.08E+08 | T | C | 0.02104 | 0.3506 | 0.0418 | 4.77E-17 | -0.04412 | 0.034565 | 0.2 | 70.35062 |
| rs1506869 | 8 | 25269102 | A | C | 0.328855 | -0.052 | 0.0091 | 1.02E-08 | 0.015034 | 0.012391 | 0.23 | 32.65281 |
| rs16991615 | 20 | 5948227 | A | G | 0.05205 | 0.1451 | 0.0265 | 4.30E-08 | -0.01312 | 0.022058 | 0.55 | 29.98056 |
| rs17033114 | 12 | 1.03E+08 | C | T | 0.125296 | -0.1322 | 0.0145 | 7.35E-20 | 0.01773 | 0.023609 | 0.450001 | 83.12345 |
| rs17116149 | 10 | 1.06E+08 | A | G | 0.038813 | 0.2688 | 0.0197 | 1.83E-42 | 0.027398 | 0.046453 | 0.56 | 186.1756 |
| rs17631680 | 2 | 67090367 | C | T | 0.101614 | -0.1045 | 0.0179 | 5.14E-09 | -0.02304 | 0.01845 | 0.21 | 34.08185 |
| rs2131371 | 12 | 46796522 | C | A | 0.731473 | 0.075 | 0.0101 | 1.20E-13 | -9.98E-05 | 0.011855 | 0.99 | 55.14123 |
| rs2207548 | 11 | 32368744 | A | C | 0.323407 | 0.1026 | 0.0103 | 1.38E-23 | 0.01262 | 0.010953 | 0.25 | 99.22404 |
| rs2270206 | 7 | 1.17E+08 | A | C | 0.204286 | 0.0577 | 0.0104 | 2.83E-08 | -0.01734 | 0.014993 | 0.25 | 30.78092 |
| rs2277339 | 12 | 57146069 | G | T | 0.132493 | -0.0801 | 0.0121 | 3.12E-11 | -0.02449 | 0.017393 | 0.16 | 43.82187 |
| rs2293607 | 3 | 1.69E+08 | C | T | 0.343688 | -0.0845 | 0.0093 | 1.61E-19 | 0.00697 | 0.012549 | 0.58 | 82.55515 |
| rs2306022 | 15 | 68628163 | T | C | 0.08676 | 0.1104 | 0.0152 | 3.89E-13 | -0.00213 | 0.019096 | 0.91 | 52.75305 |
| rs2413620 | 22 | 40668988 | G | C | 0.256586 | 0.1028 | 0.0095 | 4.35E-27 | -0.03923 | 0.013131 | 0.0028 | 117.0943 |
| rs28508285 | 9 | 92254897 | G | A | 0.181406 | 0.0735 | 0.0111 | 4.22E-11 | -0.00704 | 0.01833 | 0.7 | 43.84553 |
| rs34933909 | 5 | 1.76E+08 | T | G | 0.459616 | 0.0916 | 0.0151 | 1.44E-09 | 0.02415 | 0.010914 | 0.027 | 36.7988 |

| | | | | | | | | | | | | |
|---|---|---|---|---|---|---|---|---|---|---|---|---|
| rs3804984 | 3 | 4716214 | C | T | 0.470262 | -0.0618 | 0.0092 | 1.89E-11 | 0.011651 | 0.01139 | 0.31 | 45.123 |
| rs3820282 | 1 | 22468215 | T | C | 0.250518 | 0.1542 | 0.0102 | 4.06E-52 | -0.00363 | 0.014816 | 0.81 | 228.5415 |
| rs41308088 | 20 | 62293118 | T | C | 0.082701 | 0.12 | 0.0197 | 1.15E-09 | -0.03382 | 0.019869 | 0.089 | 37.10451 |
| rs4858590 | 3 | 24260363 | T | C | 0.434708 | 0.0553 | 0.0086 | 1.44E-10 | 0.019677 | 0.010801 | 0.068 | 41.34757 |
| rs547904 | 3 | 8947601 | G | A | 0.657141 | 0.0505 | 0.0092 | 4.86E-08 | -0.00801 | 0.012033 | 0.51 | 30.13032 |
| rs56263516 | 6 | 31413707 | G | T | 0.135612 | 0.0661 | 0.0121 | 4.20E-08 | 0.002514 | 0.018251 | 0.89 | 29.84206 |
| rs58415480 | 6 | 1.53E+08 | G | C | 0.177134 | 0.1999 | 0.0141 | 7.48E-46 | -0.02635 | 0.015067 | 0.08 | 200.9944 |
| rs62323681 | 4 | 54548347 | G | A | 0.062054 | 0.1606 | 0.0231 | 3.43E-12 | -0.06411 | 0.021778 | 0.0032 | 48.33523 |
| rs6500282 | 16 | 50138100 | C | A | 0.700054 | 0.0655 | 0.0093 | 1.96E-12 | 0.015875 | 0.01182 | 0.18 | 49.60362 |
| rs6903575 | 6 | 74503730 | A | G | 0.495939 | -0.0618 | 0.0085 | 4.50E-13 | 0.007702 | 0.01075 | 0.47 | 52.86104 |
| rs7043754 | 9 | 630424 | A | G | 0.443614 | -0.0542 | 0.0087 | 4.75E-10 | -0.0073 | 0.010798 | 0.5 | 38.81117 |
| rs7318389 | 13 | 40829568 | C | T | 0.202623 | 0.0594 | 0.0105 | 1.57E-08 | -0.00669 | 0.014111 | 0.64 | 32.00302 |
| rs73392700 | 11 | 224845 | C | G | 0.05756 | -0.2686 | 0.0175 | 4.59E-53 | 0.01196 | 0.025782 | 0.64 | 235.5768 |
| rs77687125 | 2 | 2.43E+08 | T | C | 0.398987 | 0.065 | 0.0113 | 7.57E-09 | -0.00685 | 0.010986 | 0.53 | 33.08769 |
| rs78378222 | 17 | 7571752 | G | T | 0.013713 | 0.8175 | 0.0487 | 3.95E-63 | -0.04262 | 0.050737 | 0.4 | 281.7826 |
| rs7841047 | 8 | 71265978 | A | G | 0.247413 | 0.0651 | 0.0102 | 1.96E-10 | 0.009343 | 0.014602 | 0.52 | 40.73411 |
| rs8105767 | 19 | 22215441 | G | A | 0.311329 | 0.0496 | 0.0091 | 4.97E-08 | -0.01194 | 0.011815 | 0.31 | 29.70826 |
| rs9549260 | 13 | 41254104 | A | C | 0.284865 | 0.0559 | 0.0092 | 1.53E-09 | 0.002353 | 0.012965 | 0.86 | 36.91855 |

SNP, single nucleotide polymorphism; Chr, chromosome; EAF, effect allele frequency.

**Supplementary Table 3.** Detailed information of SNPs used in the MR analysis of UF on AF.

| SNP | Chr | Position | A1 | A2 | EAF | Uterine Fibroids | | | Atrial Fibrillation | | | F-val |
|---|---|---|---|---|---|---|---|---|---|---|---|---|
| | | | | | | Beta | SE | P-val | Beta | SE | P-val | |
| rs10069690 | 5 | 1279790 | T | C | 0.262543 | 0.1226 | 0.01 | 1.79E-34 | 0.0115 | 0.008 | 0.1508 | 150.3064 |
| rs1045405 | 11 | 252733 | C | T | 0.152915 | 0.0759 | 0.0127 | 2.07E-09 | 0.0192 | 0.0177 | 0.2769 | 35.71682 |
| rs10815717 | 9 | 801571 | A | G | 0.495339 | 0.0776 | 0.009 | 6.76E-18 | 0.0013 | 0.0069 | 0.8462 | 74.34214 |
| rs10947616 | 6 | 36620499 | G | C | 0.273529 | -0.0695 | 0.0096 | 5.86E-13 | 0.008 | 0.0081 | 0.3273 | 52.41116 |
| rs11008551 | 10 | 31913890 | T | G | 0.197621 | -0.0609 | 0.011 | 3.03E-08 | 0.012 | 0.0083 | 0.1468 | 30.65109 |
| rs117245733 | 13 | 40723944 | A | G | 0.020552 | 0.3827 | 0.0379 | 6.12E-24 | -0.0477 | 0.0303 | 0.1154 | 101.9612 |
| rs11786929 | 8 | 1.3E+08 | C | T | 0.365194 | 0.0544 | 0.009 | 1.53E-09 | -5.00E-04 | 0.0071 | 0.9476 | 36.53503 |
| rs11887109 | 2 | 1E+08 | G | A | 0.525423 | 0.0593 | 0.0088 | 1.56E-11 | 7.00E-04 | 0.0067 | 0.9155 | 45.40887 |
| rs12479436 | 2 | 11666339 | G | T | 0.585117 | 0.0839 | 0.0087 | 8.06E-22 | -6.00E-04 | 0.0068 | 0.9287 | 92.99981 |
| rs12674731 | 8 | 30175381 | C | A | 0.27411 | 0.0574 | 0.0095 | 1.49E-09 | 0.0054 | 0.0078 | 0.4928 | 36.50675 |
| rs12902948 | 15 | 77849254 | C | T | 0.279137 | -0.0629 | 0.01 | 3.50E-10 | -0.0111 | 0.0072 | 0.1222 | 39.56379 |
| rs149934734 | 11 | 1.08E+08 | T | C | 0.02104 | 0.3506 | 0.0418 | 4.77E-17 | -0.0218 | 0.0244 | 0.3708 | 70.35062 |
| rs1506869 | 8 | 25269102 | A | C | 0.328855 | -0.052 | 0.0091 | 1.02E-08 | 2.00E-04 | 0.0075 | 0.9781 | 32.65281 |
| rs16991615 | 20 | 5948227 | A | G | 0.05205 | 0.1451 | 0.0265 | 4.30E-08 | -0.0181 | 0.0135 | 0.179 | 29.98056 |
| rs17033114 | 12 | 1.03E+08 | C | T | 0.125296 | -0.1322 | 0.0145 | 7.35E-20 | -0.0149 | 0.0152 | 0.3277 | 83.12345 |
| rs17116149 | 10 | 1.06E+08 | A | G | 0.038813 | 0.2688 | 0.0197 | 1.83E-42 | 0.0267 | 0.0303 | 0.3788 | 186.1756 |
| rs17631680 | 2 | 67090367 | C | T | 0.101614 | -0.1045 | 0.0179 | 5.14E-09 | -0.0098 | 0.0113 | 0.3861 | 34.08185 |
| rs2131371 | 12 | 46796522 | C | A | 0.731473 | 0.075 | 0.0101 | 1.20E-13 | 0.0118 | 0.0071 | 0.09813 | 55.14123 |
| rs2207548 | 11 | 32368744 | A | C | 0.323407 | 0.1026 | 0.0103 | 1.38E-23 | 0.0165 | 0.0068 | 0.01536 | 99.22404 |
| rs2270206 | 7 | 1.17E+08 | A | C | 0.204286 | 0.0577 | 0.0104 | 2.83E-08 | 0.0211 | 0.0093 | 0.02286 | 30.78092 |
| rs2277339 | 12 | 57146069 | G | T | 0.132493 | -0.0801 | 0.0121 | 3.12E-11 | -0.0201 | 0.011 | 0.06795 | 43.82187 |
| rs2293607 | 3 | 1.69E+08 | C | T | 0.343688 | -0.0845 | 0.0093 | 1.61E-19 | 0.0043 | 0.0078 | 0.577301 | 82.55515 |
| rs2306022 | 15 | 68628163 | T | C | 0.08676 | 0.1104 | 0.0152 | 3.89E-13 | -0.0059 | 0.0118 | 0.6134 | 52.75305 |
| rs2413620 | 22 | 40668988 | G | C | 0.256586 | 0.1028 | 0.0095 | 4.35E-27 | -0.0103 | 0.0081 | 0.1994 | 117.0943 |
| rs28508285 | 9 | 92254897 | G | A | 0.181406 | 0.0735 | 0.0111 | 4.22E-11 | 0.0101 | 0.0114 | 0.3756 | 43.84553 |
| rs34242502 | 17 | 12561985 | A | G | 0.430701 | 0.069 | 0.0106 | 6.57E-11 | 0.0037 | 0.0107 | 0.7271 | 42.3724 |
| rs34933909 | 5 | 1.76E+08 | T | G | 0.459616 | 0.0916 | 0.0151 | 1.44E-09 | 0.0209 | 0.0173 | 0.2257 | 36.7988 |

| | | | | | | | | | | | | |
|---|---|---|---|---|---|---|---|---|---|---|---|---|
| rs3804984 | 3 | 4716214 | C | T | 0.470262 | -0.0618 | 0.0092 | 1.89E-11 | 0.0038 | 0.0069 | 0.584901 | 45.123 |
| rs3820282 | 1 | 22468215 | T | C | 0.250518 | 0.1542 | 0.0102 | 4.06E-52 | 0.0118 | 0.0091 | 0.1941 | 228.5415 |
| rs41308088 | 20 | 62293118 | T | C | 0.082701 | 0.12 | 0.0197 | 1.15E-09 | 0.0196 | 0.0131 | 0.135 | 37.10451 |
| rs4858590 | 3 | 24260363 | T | C | 0.434708 | 0.0553 | 0.0086 | 1.44E-10 | -0.0168 | 0.0067 | 0.01183 | 41.34757 |
| rs547904 | 3 | 8947601 | G | A | 0.657141 | 0.0505 | 0.0092 | 4.86E-08 | -0.0118 | 0.0074 | 0.1082 | 30.13032 |
| rs56263516 | 6 | 31413707 | G | T | 0.135612 | 0.0661 | 0.0121 | 4.20E-08 | 0.008 | 0.0119 | 0.5038 | 29.84206 |
| rs58415480 | 6 | 1.53E+08 | G | C | 0.177134 | 0.1999 | 0.0141 | 7.48E-46 | -0.0048 | 0.0101 | 0.637901 | 200.9944 |
| rs62323681 | 4 | 54548347 | G | A | 0.062054 | 0.1606 | 0.0231 | 3.43E-12 | -0.0316 | 0.0132 | 0.01681 | 48.33523 |
| rs6500282 | 16 | 50138100 | C | A | 0.700054 | 0.0655 | 0.0093 | 1.96E-12 | 0.0125 | 0.0075 | 0.096139 | 49.60362 |
| rs6903575 | 6 | 74503730 | A | G | 0.495939 | -0.0618 | 0.0085 | 4.50E-13 | -0.0106 | 0.0066 | 0.1085 | 52.86104 |
| rs7043754 | 9 | 630424 | A | G | 0.443614 | -0.0542 | 0.0087 | 4.75E-10 | -0.0115 | 0.0067 | 0.08288 | 38.81117 |
| rs7318389 | 13 | 40829568 | C | T | 0.202623 | 0.0594 | 0.0105 | 1.57E-08 | -0.0037 | 0.0089 | 0.673 | 32.00302 |
| rs73392700 | 11 | 224845 | C | G | 0.05756 | -0.2686 | 0.0175 | 4.59E-53 | 0.02 | 0.0169 | 0.2382 | 235.5768 |
| rs77687125 | 2 | 2.43E+08 | T | C | 0.398987 | 0.065 | 0.0113 | 7.57E-09 | 0.0165 | 0.0075 | 0.02749 | 33.08769 |
| rs78378222 | 17 | 7571752 | G | T | 0.013713 | 0.8175 | 0.0487 | 3.95E-63 | 0.0324 | 0.0309 | 0.2941 | 281.7826 |
| rs7841047 | 8 | 71265978 | A | G | 0.247413 | 0.0651 | 0.0102 | 1.96E-10 | -0.0171 | 0.0091 | 0.06117 | 40.73411 |
| rs8105767 | 19 | 22215441 | G | A | 0.311329 | 0.0496 | 0.0091 | 4.97E-08 | 0.0196 | 0.0073 | 0.007308 | 29.70826 |
| rs9549260 | 13 | 41254104 | A | C | 0.284865 | 0.0559 | 0.0092 | 1.53E-09 | -0.02 | 0.008 | 0.01299 | 36.91855 |

SNP, single nucleotide polymorphism; Chr, chromosome; EAF, effect allele frequency.

**Supplementary Table 4.** Detailed information of SNPs used in the MR analysis of UF on HF.

| SNP | Chr | Position | A1 | A2 | EAF | Uterine Fibroids | | | Heart Failure | | | F-val |
|---|---|---|---|---|---|---|---|---|---|---|---|---|
| | | | | | | Beta | SE | P-val | Beta | SE | P-val | |
| rs10069690 | 5 | 1279790 | T | C | 0.262543 | 0.1226 | 0.01 | 1.79E-34 | -0.0216 | 0.0095 | 0.02318 | 150.3064 |
| rs1045405 | 11 | 252733 | C | T | 0.152915 | 0.0759 | 0.0127 | 2.07E-09 | 0.0019 | 0.0233 | 0.9367 | 35.71682 |
| rs10815717 | 9 | 801571 | A | G | 0.495339 | 0.0776 | 0.009 | 6.76E-18 | -0.0063 | 0.0081 | 0.4393 | 74.34214 |
| rs11008551 | 10 | 31913890 | T | G | 0.197621 | -0.0609 | 0.011 | 3.03E-08 | 0.0078 | 0.0097 | 0.4219 | 30.65109 |
| rs117245733 | 13 | 40723944 | A | G | 0.020552 | 0.3827 | 0.0379 | 6.12E-24 | 0.0274 | 0.0316 | 0.3869 | 101.9612 |
| rs11786929 | 8 | 1.3E+08 | C | T | 0.365194 | 0.0544 | 0.009 | 1.53E-09 | 0.0097 | 0.0085 | 0.254 | 36.53503 |
| rs11887109 | 2 | 1E+08 | G | A | 0.525423 | 0.0593 | 0.0088 | 1.56E-11 | 0.0061 | 0.0081 | 0.4523 | 45.40887 |
| rs12479436 | 2 | 11666339 | G | T | 0.585117 | 0.0839 | 0.0087 | 8.06E-22 | -0.0012 | 0.008 | 0.8849 | 92.99981 |
| rs12674731 | 8 | 30175381 | C | A | 0.27411 | 0.0574 | 0.0095 | 1.49E-09 | 0.0085 | 0.0093 | 0.3619 | 36.50675 |
| rs12902948 | 15 | 77849254 | C | T | 0.279137 | -0.0629 | 0.01 | 3.50E-10 | -4.00E-04 | 0.0085 | 0.9607 | 39.56379 |
| rs149934734 | 11 | 1.08E+08 | T | C | 0.02104 | 0.3506 | 0.0418 | 4.77E-17 | -0.0276 | 0.0276 | 0.3171 | 70.35062 |
| rs1506869 | 8 | 25269102 | A | C | 0.328855 | -0.052 | 0.0091 | 1.02E-08 | -0.0079 | 0.0088 | 0.3733 | 32.65281 |
| rs16991615 | 20 | 5948227 | A | G | 0.05205 | 0.1451 | 0.0265 | 4.30E-08 | -0.0059 | 0.016 | 0.710901 | 29.98056 |
| rs17033114 | 12 | 1.03E+08 | C | T | 0.125296 | -0.1322 | 0.0145 | 7.35E-20 | 0.0087 | 0.018 | 0.626299 | 83.12345 |
| rs17116149 | 10 | 1.06E+08 | A | G | 0.038813 | 0.2688 | 0.0197 | 1.83E-42 | 0.036 | 0.0353 | 0.3082 | 186.1756 |
| rs17631680 | 2 | 67090367 | C | T | 0.101614 | -0.1045 | 0.0179 | 5.14E-09 | -0.0231 | 0.0132 | 0.079811 | 34.08185 |
| rs2131371 | 12 | 46796522 | C | A | 0.731473 | 0.075 | 0.0101 | 1.20E-13 | 0.0142 | 0.0083 | 0.0879 | 55.14123 |
| rs2207548 | 11 | 32368744 | A | C | 0.323407 | 0.1026 | 0.0103 | 1.38E-23 | 0.0045 | 0.0081 | 0.5799 | 99.22404 |
| rs2270206 | 7 | 1.17E+08 | A | C | 0.204286 | 0.0577 | 0.0104 | 2.83E-08 | 0.0071 | 0.011 | 0.5201 | 30.78092 |
| rs2277339 | 12 | 57146069 | G | T | 0.132493 | -0.0801 | 0.0121 | 3.12E-11 | -0.0143 | 0.0133 | 0.2837 | 43.82187 |
| rs2293607 | 3 | 1.69E+08 | C | T | 0.343688 | -0.0845 | 0.0093 | 1.61E-19 | -3.00E-04 | 0.0093 | 0.9713 | 82.55515 |
| rs2306022 | 15 | 68628163 | T | C | 0.08676 | 0.1104 | 0.0152 | 3.89E-13 | 0.0049 | 0.0138 | 0.7218 | 52.75305 |
| rs28508285 | 9 | 92254897 | G | A | 0.181406 | 0.0735 | 0.0111 | 4.22E-11 | 0.0033 | 0.0138 | 0.811 | 43.84553 |
| rs34242502 | 17 | 12561985 | A | G | 0.430701 | 0.069 | 0.0106 | 6.57E-11 | 0.0149 | 0.012 | 0.2141 | 42.3724 |
| rs34933909 | 5 | 1.76E+08 | T | G | 0.459616 | 0.0916 | 0.0151 | 1.44E-09 | -0.0014 | 0.0079 | 0.8576 | 36.7988 |
| rs3804984 | 3 | 4716214 | C | T | 0.470262 | -0.0618 | 0.0092 | 1.89E-11 | 0.0023 | 0.0082 | 0.778099 | 45.123 |
| rs3820282 | 1 | 22468215 | T | C | 0.250518 | 0.1542 | 0.0102 | 4.06E-52 | 0.0104 | 0.0105 | 0.3252 | 228.5415 |

| | | | | | | | | | | | | |
|---|---|---|---|---|---|---|---|---|---|---|---|---|
| rs41308088 | 20 | 62293118 | T | C | 0.082701 | 0.12 | 0.0197 | 1.15E-09 | -0.0164 | 0.0152 | 0.2792 | 37.10451 |
| rs4858590 | 3 | 24260363 | T | C | 0.434708 | 0.0553 | 0.0086 | 1.44E-10 | -0.0033 | 0.0079 | 0.673301 | 41.34757 |
| rs547904 | 3 | 8947601 | G | A | 0.657141 | 0.0505 | 0.0092 | 4.86E-08 | -0.0024 | 0.0088 | 0.7886 | 30.13032 |
| rs56263516 | 6 | 31413707 | G | T | 0.135612 | 0.0661 | 0.0121 | 4.20E-08 | 0.0221 | 0.0128 | 0.084491 | 29.84206 |
| rs62323681 | 4 | 54548347 | G | A | 0.062054 | 0.1606 | 0.0231 | 3.43E-12 | -0.0159 | 0.0152 | 0.2975 | 48.33523 |
| rs6500282 | 16 | 50138100 | C | A | 0.700054 | 0.0655 | 0.0093 | 1.96E-12 | -0.0022 | 0.0089 | 0.804 | 49.60362 |
| rs6903575 | 6 | 74503730 | A | G | 0.495939 | -0.0618 | 0.0085 | 4.50E-13 | 0.0129 | 0.0078 | 0.1002 | 52.86104 |
| rs7043754 | 9 | 630424 | A | G | 0.443614 | -0.0542 | 0.0087 | 4.75E-10 | 0.0015 | 0.0078 | 0.8486 | 38.81117 |
| rs7318389 | 13 | 40829568 | C | T | 0.202623 | 0.0594 | 0.0105 | 1.57E-08 | -0.0207 | 0.0107 | 0.05259 | 32.00302 |
| rs77687125 | 2 | 2.43E+08 | T | C | 0.398987 | 0.065 | 0.0113 | 7.57E-09 | 0.0059 | 0.0087 | 0.4985 | 33.08769 |
| rs78378222 | 17 | 7571752 | G | T | 0.013713 | 0.8175 | 0.0487 | 3.95E-63 | -0.0203 | 0.0337 | 0.5465 | 281.7826 |
| rs7841047 | 8 | 71265978 | A | G | 0.247413 | 0.0651 | 0.0102 | 1.96E-10 | 0.0067 | 0.0109 | 0.538801 | 40.73411 |
| rs8105767 | 19 | 22215441 | G | A | 0.311329 | 0.0496 | 0.0091 | 4.97E-08 | -0.001 | 0.0085 | 0.9066 | 29.70826 |
| rs9549260 | 13 | 41254104 | A | C | 0.284865 | 0.0559 | 0.0092 | 1.53E-09 | 0.0164 | 0.0095 | 0.085181 | 36.91855 |

SNP, single nucleotide polymorphism; Chr, chromosome; EAF, effect allele frequency.

**Supplementary Table 5.** Detailed information of SNPs used in the MR analysis of UF on CES.

| SNP | Chr | Position | A1 | A2 | EAF | Uterine Fibroids | | | Cardioembolic Stroke | | | F-val |
|---|---|---|---|---|---|---|---|---|---|---|---|---|
| | | | | | | **Beta** | **SE** | **P-val** | **Beta** | **SE** | **P-val** | |
| rs10069690 | 5 | 1279790 | T | C | 0.262543 | 0.1226 | 0.01 | 1.79E-34 | 0.057 | 0.0256 | 0.02594 | 150.3064 |
| rs1045405 | 11 | 252733 | C | T | 0.152915 | 0.0759 | 0.0127 | 2.07E-09 | 0.0695 | 0.06 | 0.2468 | 35.71682 |
| rs10815717 | 9 | 801571 | A | G | 0.495339 | 0.0776 | 0.009 | 6.76E-18 | 0.0141 | 0.0204 | 0.491501 | 74.34214 |
| rs10947616 | 6 | 36620499 | G | C | 0.273529 | -0.0695 | 0.0096 | 5.86E-13 | 0.0023 | 0.0236 | 0.9208 | 52.41116 |
| rs11008551 | 10 | 31913890 | T | G | 0.197621 | -0.0609 | 0.011 | 3.03E-08 | -0.0134 | 0.024 | 0.5755 | 30.65109 |
| rs117245733 | 13 | 40723944 | A | G | 0.020552 | 0.3827 | 0.0379 | 6.12E-24 | 0.1001 | 0.0869 | 0.2497 | 101.9612 |
| rs11786929 | 8 | 1.3E+08 | C | T | 0.365194 | 0.0544 | 0.009 | 1.53E-09 | 0.0026 | 0.0202 | 0.8962 | 36.53503 |
| rs11887109 | 2 | 1E+08 | G | A | 0.525423 | 0.0593 | 0.0088 | 1.56E-11 | 0.0313 | 0.0189 | 0.09725 | 45.40887 |
| rs12479436 | 2 | 11666339 | G | T | 0.585117 | 0.0839 | 0.0087 | 8.06E-22 | 0.0039 | 0.0199 | 0.845 | 92.99981 |
| rs12674731 | 8 | 30175381 | C | A | 0.27411 | 0.0574 | 0.0095 | 1.49E-09 | 0.0387 | 0.0224 | 0.084619 | 36.50675 |
| rs12902948 | 15 | 77849254 | C | T | 0.279137 | -0.0629 | 0.01 | 3.50E-10 | 0.014 | 0.0205 | 0.4931 | 39.56379 |
| rs149934734 | 11 | 1.08E+08 | T | C | 0.02104 | 0.3506 | 0.0418 | 4.77E-17 | 0.0648 | 0.0683 | 0.3428 | 70.35062 |
| rs1506869 | 8 | 25269102 | A | C | 0.328855 | -0.052 | 0.0091 | 1.02E-08 | -0.0307 | 0.0219 | 0.1601 | 32.65281 |
| rs16991615 | 20 | 5948227 | A | G | 0.05205 | 0.1451 | 0.0265 | 4.30E-08 | -0.0218 | 0.0416 | 0.6009 | 29.98056 |
| rs17033114 | 12 | 1.03E+08 | C | T | 0.125296 | -0.1322 | 0.0145 | 7.35E-20 | -0.0221 | 0.0492 | 0.6534 | 83.12345 |
| rs17116149 | 10 | 1.06E+08 | A | G | 0.038813 | 0.2688 | 0.0197 | 1.83E-42 | 0.1533 | 0.0952 | 0.1072 | 186.1756 |
| rs17631680 | 2 | 67090367 | C | T | 0.101614 | -0.1045 | 0.0179 | 5.14E-09 | -0.0119 | 0.033 | 0.7181 | 34.08185 |
| rs2131371 | 12 | 46796522 | C | A | 0.731473 | 0.075 | 0.0101 | 1.20E-13 | 0.0176 | 0.0202 | 0.3846 | 55.14123 |
| rs2207548 | 11 | 32368744 | A | C | 0.323407 | 0.1026 | 0.0103 | 1.38E-23 | -0.0094 | 0.0172 | 0.587 | 99.22404 |
| rs2270206 | 7 | 1.17E+08 | A | C | 0.204286 | 0.0577 | 0.0104 | 2.83E-08 | -2.00E-04 | 0.0269 | 0.9954 | 30.78092 |
| rs2277339 | 12 | 57146069 | G | T | 0.132493 | -0.0801 | 0.0121 | 3.12E-11 | -0.0217 | 0.0341 | 0.524901 | 43.82187 |
| rs2293607 | 3 | 1.69E+08 | C | T | 0.343688 | -0.0845 | 0.0093 | 1.61E-19 | 0.0345 | 0.0226 | 0.1277 | 82.55515 |
| rs2306022 | 15 | 68628163 | T | C | 0.08676 | 0.1104 | 0.0152 | 3.89E-13 | 0.016 | 0.0366 | 0.661301 | 52.75305 |
| rs2413620 | 22 | 40668988 | G | C | 0.256586 | 0.1028 | 0.0095 | 4.35E-27 | -0.0092 | 0.0234 | 0.693999 | 117.0943 |
| rs28508285 | 9 | 92254897 | G | A | 0.181406 | 0.0735 | 0.0111 | 4.22E-11 | 0.0222 | 0.0337 | 0.51 | 43.84553 |
| rs34242502 | 17 | 12561985 | A | G | 0.430701 | 0.069 | 0.0106 | 6.57E-11 | -3.00E-04 | 0.0213 | 0.9899 | 42.3724 |
| rs34933909 | 5 | 1.76E+08 | T | G | 0.459616 | 0.0916 | 0.0151 | 1.44E-09 | 0.0301 | 0.0196 | 0.125 | 36.7988 |

| | | | | | | | | | | | | | |
|---|---|---|---|---|---|---|---|---|---|---|---|---|---|
| rs3804984 | 3 | 4716214 | C | T | 0.470262 | -0.0618 | 0.0092 | 1.89E-11 | -9.00E-04 | 0.02 | 0.966 | 45.123 |
| rs3820282 | 1 | 22468215 | T | C | 0.250518 | 0.1542 | 0.0102 | 4.06E-52 | -0.041 | 0.027 | 0.1288 | 228.5415 |
| rs41308088 | 20 | 62293118 | T | C | 0.082701 | 0.12 | 0.0197 | 1.15E-09 | 0.0931 | 0.0394 | 0.01806 | 37.10451 |
| rs4858590 | 3 | 24260363 | T | C | 0.434708 | 0.0553 | 0.0086 | 1.44E-10 | 0.0019 | 0.0193 | 0.9221 | 41.34757 |
| rs547904 | 3 | 8947601 | G | A | 0.657141 | 0.0505 | 0.0092 | 4.86E-08 | -0.0161 | 0.0212 | 0.4481 | 30.13032 |
| rs56263516 | 6 | 31413707 | G | T | 0.135612 | 0.0661 | 0.0121 | 4.20E-08 | 0.1108 | 0.0388 | 0.004344 | 29.84206 |
| rs58415480 | 6 | 1.53E+08 | G | C | 0.177134 | 0.1999 | 0.0141 | 7.48E-46 | 0.0132 | 0.0271 | 0.6269 | 200.9944 |
| rs62323681 | 4 | 54548347 | G | A | 0.062054 | 0.1606 | 0.0231 | 3.43E-12 | -0.0677 | 0.0385 | 0.078421 | 48.33523 |
| rs6500282 | 16 | 50138100 | C | A | 0.700054 | 0.0655 | 0.0093 | 1.96E-12 | 0.0117 | 0.0217 | 0.5914 | 49.60362 |
| rs6903575 | 6 | 74503730 | A | G | 0.495939 | -0.0618 | 0.0085 | 4.50E-13 | -0.0174 | 0.0189 | 0.3569 | 52.86104 |
| rs7043754 | 9 | 630424 | A | G | 0.443614 | -0.0542 | 0.0087 | 4.75E-10 | -0.0389 | 0.019 | 0.04021 | 38.81117 |
| rs7318389 | 13 | 40829568 | C | T | 0.202623 | 0.0594 | 0.0105 | 1.57E-08 | -0.0056 | 0.0268 | 0.8336 | 32.00302 |
| rs73392700 | 11 | 224845 | C | G | 0.05756 | -0.2686 | 0.0175 | 4.59E-53 | -0.0815 | 0.0553 | 0.1406 | 235.5768 |
| rs77687125 | 2 | 2.43E+08 | T | C | 0.398987 | 0.065 | 0.0113 | 7.57E-09 | 0.044 | 0.0257 | 0.086521 | 33.08769 |
| rs78378222 | 17 | 7571752 | G | T | 0.013713 | 0.8175 | 0.0487 | 3.95E-63 | -0.0458 | 0.1085 | 0.6732 | 281.7826 |
| rs7841047 | 8 | 71265978 | A | G | 0.247413 | 0.0651 | 0.0102 | 1.96E-10 | -0.0153 | 0.0263 | 0.5616 | 40.73411 |
| rs8105767 | 19 | 22215441 | G | A | 0.311329 | 0.0496 | 0.0091 | 4.97E-08 | -0.0121 | 0.0212 | 0.569399 | 29.70826 |
| rs9549260 | 13 | 41254104 | A | C | 0.284865 | 0.0559 | 0.0092 | 1.53E-09 | 0.0467 | 0.0231 | 0.04342 | 36.91855 |

SNP, single nucleotide polymorphism; Chr, chromosome; EAF, effect allele frequency.
