# Supplementary material for: Causal relationship between uterine fibroids and cardiovascular disease: A two-sample Mendelian randomization study
Source: Medicine (Baltimore). 2025 Feb 28;104(9):e41713. doi: 10.1097/MD.0000000000041713 (PMC11875593; doi:10.1097/MD.0000000000041713)
Supplement: Supplementary file 2 [file medi-104-e41713-s002.pdf]

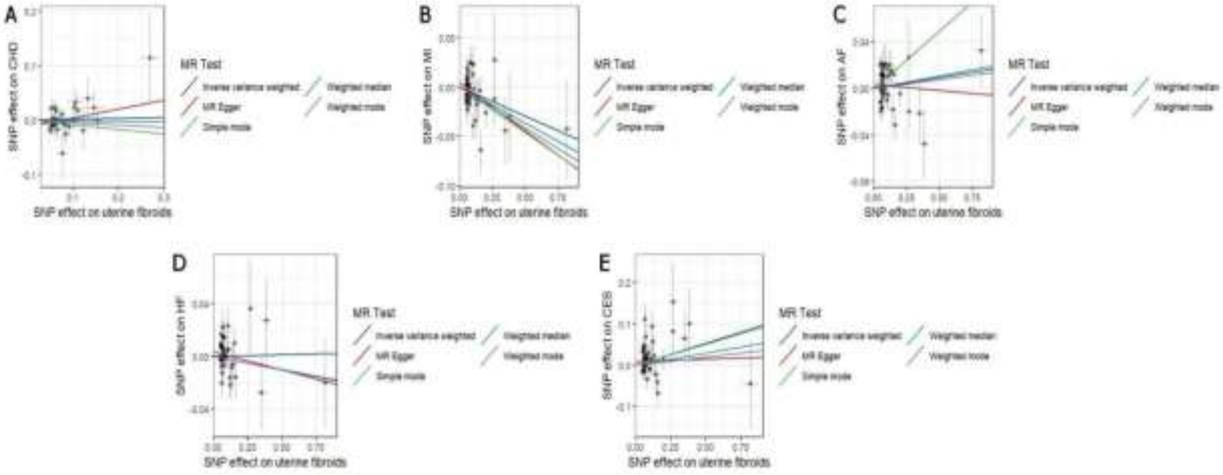

Supplementary Figure 1. Scatter plot. (**A**) uterine fibroids and coronary heart disease; (**B**) uterine fibroids and myocardial infarction; (**C**) uterine fibroids and atrial fibrillation; (**D**) uterine fibroids and heart failure; (**E**) uterine fibroids and cardioembolic stroke.

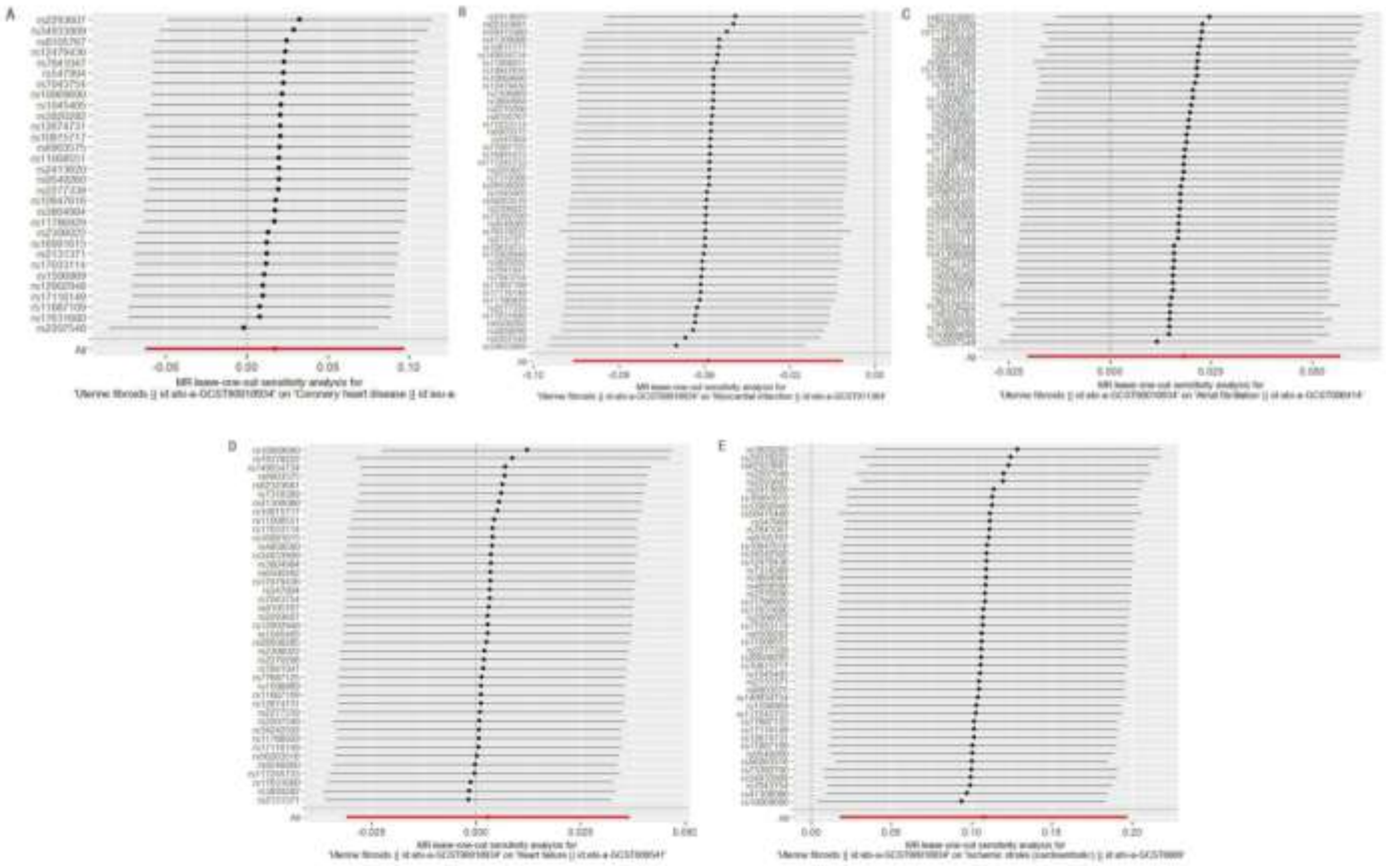

Supplementary Figure 2. Leave-one-out sensitivity analysis. (**A**) uterine fibroids and coronary heart disease; (**B**) uterine fibroids and myocardial infarction; (**C**) uterine fibroids and atrial fibrillation; (**D**) uterine fibroids and heart failure; (**E**) uterine fibroids and cardioembolic stroke.

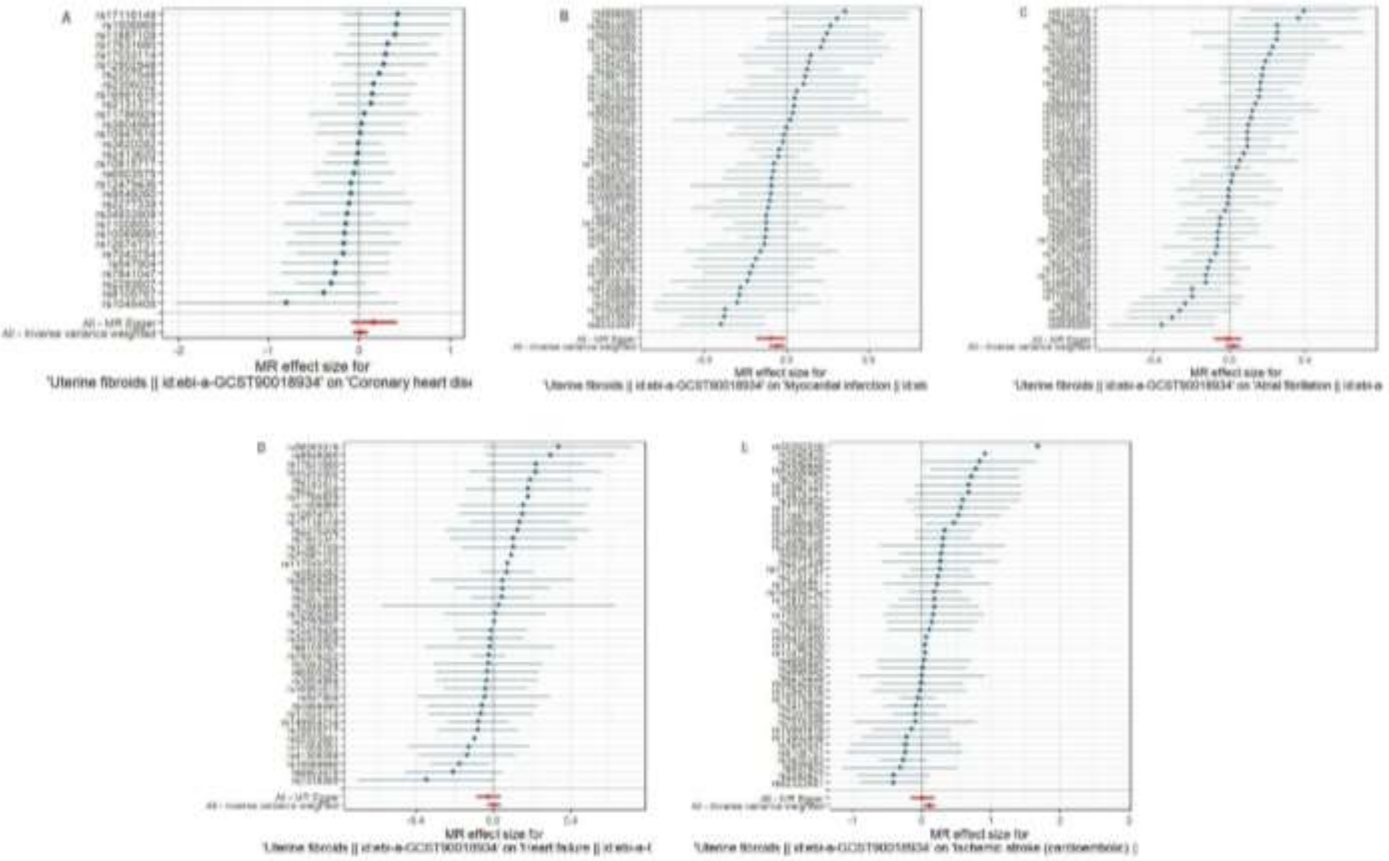

Supplementary Figure 3. Forest plot. (**A**) uterine fibroids and coronary heart disease; (**B**) uterine fibroids and myocardial infarction; (**C**) uterine fibroids and atrial fibrillation; (**D**) uterine fibroids and heart failure; (**E**) uterine fibroids and cardioembolic stroke.

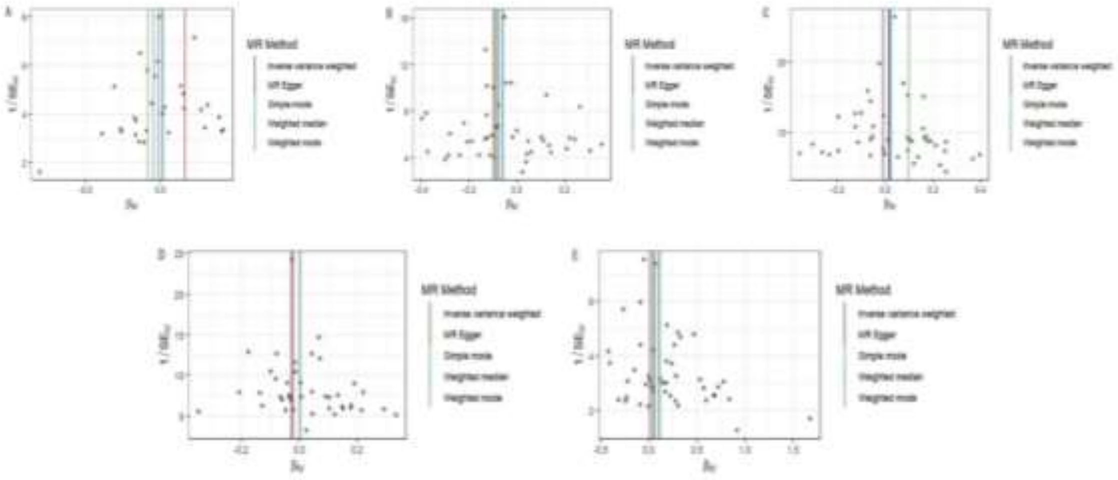

Supplementary Figure 4. Funnel plot. (**A**) uterine fibroids and coronary heart disease; (**B**) uterine fibroids and myocardial infarction; (**C**) uterine fibroids and atrial fibrillation; (**D**) uterine fibroids and heart failure; (**E**) uterine fibroids and cardioembolic stroke.
